# Characterization of a panel of six β_2_-adrenergic receptor antibodies by indirect immunofluorescence microscopy

**DOI:** 10.1186/1465-9921-9-32

**Published:** 2008-04-18

**Authors:** Yulia A Koryakina, Tristan W Fowler, Stacie M Jones, Bradley J Schnackenberg, Lawrence E Cornett, Richard C Kurten

**Affiliations:** 1 Department of Physiology and Biophysics, University of Arkansas for Medical Sciences, Little Rock, AR 72205, USA; 2 Department of Pediatrics, University of Arkansas for Medical Sciences, AR, USA; 3 Arkansas Children's Hospital Research Institute, Little Rock, AR 72202, USA

## Abstract

**Background:**

The β_2_-adrenergic receptor (β_2_AR) is a primary target for medications used to treat asthma. Due to the low abundance of β_2_AR, very few studies have reported its localization in tissues. However, the intracellular location of β_2_AR in lung tissue, especially in airway smooth muscle cells, is very likely to have a significant impact on how the airways respond to β-agonist medications. Thus, a method for visualizing β_2_AR in tissues would be of utility. The purpose of this study was to develop an immunofluorescent labeling technique for localizing native and recombinant β_2_AR in primary cell cultures.

**Methods:**

A panel of six different antibodies were evaluated in indirect immunofluorescence assays for their ability to recognize human and rat β_2_AR expressed in HEK 293 cells. Antibodies capable of recognizing rat β_2_AR were identified and used to localize native β_2_AR in primary cultures of rat airway smooth muscle and epithelial cells. β_2_AR expression was confirmed by performing ligand binding assays using the β-adrenergic antagonist [3H] dihydroalprenolol ^([3H]DHA)^.

**Results:**

Among the six antibodies tested, we identified three of interest. An antibody developed against the C-terminal 15 amino acids of the human β_2_AR (Ab-Bethyl) specifically recognized human but not rat β_2_AR. An antibody developed against the C-terminal domain of the mouse β_2_AR (Ab-sc570) specifically recognized rat but not human β_2_AR. An antibody developed against 78 amino acids of the C-terminus of the human β_2_AR (Ab-13989) was capable of recognizing both rat and human β_2_ARs. In HEK 293 cells, the receptors were predominantly localized to the cell surface. By contrast, about half of the native rat β_2_AR that we visualized in primary cultures of rat airway epithelial and smooth muscle cells using Ab-sc570 and Ab-13989 was found inside cells rather than on their surface.

**Conclusion:**

Antibodies have been identified that recognize human β_2_AR, rat β_2_AR or both rat and human β_2_AR. Interestingly, the pattern of expression in transfected cells expressing millions of receptors was dramatically different from that in primary cell cultures expressing only a few thousand native receptors. We anticipate that these antibodies will provide a valuable tool for evaluating the expression and trafficking of β_2_AR in tissues.

## Introduction

The β_2_-adrenergic receptor (β_2_AR) is found in several cell types within the lung where it mediates a number of important functions including relaxation of airway smooth muscle [1-3], activation of ion and fluid transport in epithelial cells [4], inhibition of mediator release from mast cells [5], stimulation of surfactant secretion in alveolar type 2 cells and stimulation of mucus secretion by submucosal glands [6-8]. The β_2_AR in smooth muscle cells is thought to be the principal target for the β-agonist medications used to treat asthma and other obstructive airway diseases. Activation of the β_2_AR by β-agonists like albuterol or salbutamol is capable of inhibiting (bronchoprotection) or reversing (bronchodilation) contractile processes.

Continuous β-agonist exposure results in tolerance to their bronchodilating effects. The problem of tolerance may pose risks to patients using both short-acting (SABA) and long-acting beta-agonists medications (LABAs). The LABA medications were developed as controller medications. However, in 2005 the U.S. FDA issued a Public Health Advisory stating that the use of LABAs might increase the risk of severe asthma episodes (and death) and advised against the use of LABAs as the first line, monotherapy for the treatment of asthma. It is thought that this clinical tolerance is the result of cellular mechanisms used to attenuate the cellular responses to β-agonist activation of β_2_AR.

The β_2_AR is a prototypical G-protein coupled receptor containing seven transmembrane α-helical regions. The N-terminal domain and three loops are located on the extracellular face of the plasma membrane, and the C-terminal domain and three loops are also located on the intracellular (or cytoplasmic) face of the plasma membrane [9]. When activated by ligand binding, β_2_ARs couple via the third intracellular loop to a heterotrimeric stimulatory G_s_-protein resulting in G_sα _subunit dissociation, GTP binding, and adenylyl cyclase activation. This occurs within seconds of ligand binding, and the resulting elevation in intracellular cAMP levels is responsible for the relaxation of airway smooth muscle leading to bronchodilation [10,2].

Bronchodilatory responses are of limited duration because sustained activation of β_2_AR is accompanied by receptor phosphorylation and by the binding of β-arrestin, thereby inhibiting further interaction and activation of G_s_. These events lead to desensitization. β-arrestin also binds coated pit components like AP-2 and clathrin, thereby resulting in endocytosis and a loss in the number of receptors on the cell surface. Thus, both short-term and long-term mechanisms exist for attenuating β_2_AR signalling [11].

The recovery in the number of receptors on plasma membrane following endocytosis is largely accomplished by recycling of the intracellular receptors back to the surface. Prolonged or chronic exposure to β-agonists causes trafficking of the receptors to lysosomes and subsequent degradation and loss of the receptors [12,13]. Much of the intricate regulatory mechanisms involved in β_2_AR signalling have been defined by using cultured cell lines and recombinant, epitope-tagged receptors expressed at levels much higher than normal. We think that it is important to determine if the mechanisms defined in engineered cell lines are also operational in cells present in a normal physiological setting. Unfortunately, immunological reagents useful for detecting native β_2_AR in tissues have not been carefully characterized. We have used indirect immunfluorescence microscopy to evaluate a panel of six antibodies for use in visualizing rat and human β_2_AR in transfected HEK 293 cells and in primary cultures of rat airway epithelial and smooth muscle cells. Our studies indicate that the level of receptor expression may have an impact on the location of receptors within cells.

## Methods

### Cell Culture, Plasmids and Transfection

The human embryonic cell line, HEK 293, was maintained in Dulbecco's modified Eagle's medium/Ham's F12 (50:50) (Cellgro, Herndon, VA) supplemented with 5% calf serum, 1% antibiotic/antimycotic in a 5% CO_2 _incubator at 37°C. HEK 293 cells stably expressing human β_2_AR [14] were maintained in media containing 200 μg/ml G418 (Cellgro). The expression plasmid pExpress1-ratβ_2_-AR was purchased from ATCC. Cells were transiently transfected with pExpress1-ratβ_2_-AR (1 μg/35 mm dish) using the calcium phosphate precipitation method [15,16]. A cDNA encoding human β_2_AR was fused to the N-terminus of pEYFP-N1 (Clontech, Mountain View, CA) [14,17].

### Receptor binding assay on intact cells

Cell monolayers were lifted with cold PBS supplemented with 5 mM EDTA using a rubber policeman and washed twice with PBS by centrifugation. Approximately 1.2 × 10^6 ^cells/ml were incubated in triplicate with a single saturating concentration of [^3^H]Dihydroalprenolol (DHA) (~5 nM) (PerkinElmer, Boston, MA; specific activity = 117.8 Ci/mmol) for 20 minutes at 30°C. Incubations were terminated by vacuum filtration through glass fiber filters presoaked in assay buffer (50 mM Tris, 2 mM MgCl_2_, pH 7.4) and repeated washes with ice-cold assay buffer. Bound radioactivity was determined by scintillation counting. Nonspecific binding was determined by using 0.1 μM (-)-propranolol (Sigma, St. Louis, MO).

### Primary Rat Airway Cell Cultures

The transportation, care, and use of animals for the reported studies was in accordance with the Animal Welfare Act (7 U.S.C. et seq.) and other applicable federal laws, guidelines, and policies. The procedures for handling animals were approved by the Institutional Animal Care and Use Committee of the University of Arkansas for Medical Sciences. Adult female Sprague-Dawley rats (250 g) were euthanized by intraperitoneal injection of Euthasol (0.22 ml/kg). The chest cavity was opened and the trachea and lungs were dissected out and transferred to a dish containing PBS.

Airway smooth muscle cells (ASMC) were generated from explants of excised tracheas. The entire trachea between the larynx and main stem bronchi was removed and placed in a sterile dish containing PBS supplemented with a 2% antibiotic/antimycotic. After additional surrounding tissue was removed with the aid of a dissecting microscope, the tracheal segment was split longitudinally and dissected into 2–3 mm squares. All segments from a single trachea were then placed with the intima side down in separate sterile 35 mm dishes. The explants were incubated in a 5% CO_2 _incubator at 37°C. After allowing the explants to adhere, 2 ml of DMEM/F12, 20% calf serum, 2% antibiotic-antimycotic was added to cover the explants. Once cells became locally confluent, the serum concentration was reduced to 10%. Media was changed every other day before confluency was achieved (~3 weeks), at which point the tracheal explants were removed.

Rat airway epithelial cell cultures were prepared by intrapulmonary enzyme digestion as follows. Excised lungs were cleared of blood by perfusing PBS (~25 ml) through the pulmonary arteries. The airways were then flushed four times with calcium- and magnesium-free Dulbecco's PBS via the trachea (~40 ml), filled with a microbially produced trypsin-like enzyme (TrypLE, Gibco Invitrogen). The trachea was clamped, and the lung was incubated at 37°C for 75 minutes. Following the intrapulmonary digestion, the airways were washed twice with DMEM/F12, 5% calf serum (~25 ml total) and twice with PBS (~25 ml) to flush out epithelial plaques. The plaques were collected by centrifugation at 900 g for 8 minutes. The pellet was resuspended in DMEM/F12, 5% calf serum and aliquots were cultured on plastic dishes in a 5% CO_2 _incubator at 37°C for up to one week.

### Indirect Immunofluorescence Microscopy

For indirect immunofluorescence microscopy, HEK 293 cells were grown on glass coverslips and treated with or without 10 μM isoproterenol for 4.5 hours. Cells were fixed with freshly prepared 3.6% paraformaldehyde in PBS, blocked and permeabilized in PBS containing 1% BSA, 5% serum and 0.1% Triton X-100. β_2_AR were visualized using the labeled avidin-biotin method. Samples were incubated with primary antibody followed by separate incubations with biotinylated secondary antibody and with Texas-Red labeled Avidin D (Vector Laboratories Inc., Burlingame, CA). Optimal dilutions of the antibodies were determined in titration experiments. Antibodies were diluted in the permeabilization buffer and samples washed with PBS after each incubation. The nuclei were stained with 30 nM 4,6-diamidinophenylindole (DAPI). Antibody dilutions were as follows: Ab-Bethyl (rabbit polyclonal antipeptide antibody, Bethyl Laboratories Inc., Montgomery, TX), 1:50; Ab-sc570 (rabbit polyclonal antipeptide antibody, Santa Cruz Biotechnology, Santa Cruz, CA), 1:300; Ab-13989 (chicken polyclonal antibody, Abcam, Inc., Cambridge, MA), 1:300. Secondary goat anti-rabbit (Vector Laboratories, Inc.) and rabbit anti-chicken (ab6752, Abcam, Inc.) biotinylated antibodies were used at a dilution of 1:200. Sc569 antibody was from Santa Cruz Biotechnology, IMG-71135 was from Imgenex Corporation, San Diego, CA, and ab13300 was purchased from Abcam.

A similar protocol was used for localization of the endogenous β_2_AR in primary cultures of rat airway smooth muscle cells (ASMC) and rat airway epithelial cells (AEC) except that the samples were double-labeled with β_2_AR and cell-type specific marker antibodies. Ab-sc570 and Ab-13989 antibodies were used at a dilution of 1:250. Mouse monoclonal anti-smooth muscle alpha-actin antibody (ab18460, Abcam, Inc.) and mouse monoclonal anti-E-cadherin (BD Transduction Labs, Franklin Lakes, NJ) were used at a dilution of 1:100. Donkey anti-mouse FITC-conjugated secondary antibody (Jackson ImmunoResearch Laboratories, Inc., West Grove, PA) was used at a dilution of 1:250. Ab-sc570 specificity was determined by preincubating the antibody with a five-fold (by weight) excess of blocking peptide (sc570p, Santa Cruz Biotechnology) for 2 hours at room temperature prior to dilution in buffer for indirect immunofluorescence as described above.

All samples were mounted in Fluoromount-G mounting medium (Electron Microscopy Sciences, Hatfield, PA) and visualized by epifluorescence (Axioskop 2 plus microscope, Carl Zeiss Inc., Thornwood, NY) and confocal microscopy (LSM510 Axiovert 200 M confocal microscope, Carl Zeiss Inc.) using a Zeiss Plan-Apo 63× 1.40NA oil immersion objective.

The acquisition settings were kept constant between specimens. Images were stored as a tagged image format.

### Data Analysis/Statistical Methods

In radioligand binding experiments, [^3^H]DHA binding to cells at each time point was measured in triplicate. Each "n" represented data from one set of cell culture plates (one condition). To achieve statistical significance, experiments were performed at n = 4. Data are presented as the mean ± S.E.M. A group t-test was used with p < 0.05 accepted as significant.

## Results and Discussion

### Ab-Bethyl Specifically Recognizes Human β_2_AR in HEK 293 Cells

HEK 293 cells express low level of endogenous β_2_AR [13]. In our experiments, we used HEK 293 cells stably and transiently expressing human and rat β_2_AR, respectively. Receptor expression and cellular location was determined using indirect immunofluorescence microscopy. A labeled avidin-biotin method was used to enhance sensitivity (approximately four-fold greater sensitivity than labeled secondary antibodies alone). Using this approach, six different β_2_AR antibodies were tested for their ability to recognize human and rat β_2_AR in HEK 293 cells (Table 1). Three antibodies (Sc569, raised against the C-terminal domain of the human β_2_AR; IMG-71135, and ab13300, each raised against the N-terminal domain of the human β_2_AR) recognized neither rat nor human β_2_AR in HEK 293 cells.

**Table 1:** Antibodies Used for IIF on HEK 293 Cells Expressing Human and Rat β_2_AR

Antibody	Source	Immunogen	Human β_2_-AR	Rat β_2_-AR
Sc569	Santa Cruz Biotechnology	C-terminal domain of human β_2_AR	-	-
IMG-71135	Imgenex Corporation	N-terminal domain of human β_2_AR	-	-
Ab13300	Abcam, Inc.	N-terminal domain of human β_2_AR	-	-
Ab-Bethyl	Bethyl Laboratories, Inc.	Last 15 aa of C-terminal domain of human β_2_AR	+	-
Ab-sc570	Santa Cruz Biotechnology	C-terminal domain of mouse β_2_AR	-	+
Ab-13989	Abcam, Inc.	78 aa of the C-terminus of the human β_2_AR	+	+

"-" and "+" indicate absence and presence of the signal

**Figure 1:**
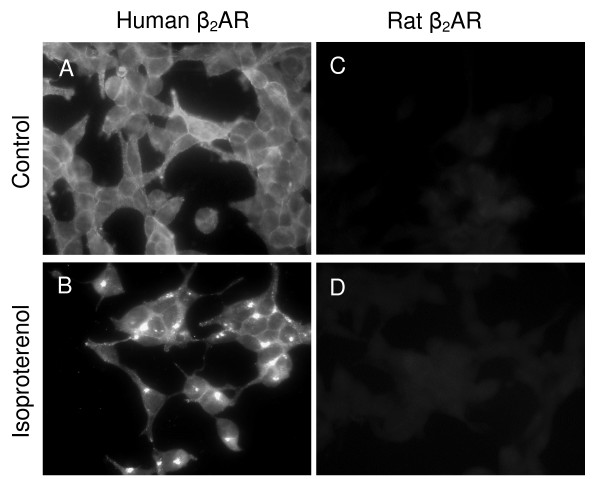
**Ab-Bethyl Specifically Recognizes Human β_2_AR in HEK 293 Cells**. HEK 293 cells stably expressing human β_2_AR (A, B) and HEK 293 cells transiently expressing rat β_2_AR (C, D) were either untreated (A, C) or treated (B, D) with isoproterenol for 4.5 h in parallel, fixed and processed for microscopy (Axioskop 2 plus epifluorescent microscope).

Ab-Bethyl (raised against the last 15 amino acids of the C-terminus of the human β_2_AR) recognized human β_2_AR at a dilution of 1:50 in HEK 293 cells stably expressing human β_2_AR (Figure 1A and 1B). In untreated cells, the receptors were predominantly localized to the cell surface (Figure 1A); whereas, after isoproterenol treatment, receptors were localized to vesicles within the cells (Figure 1B), consistent with receptor internalization. Ab-Bethyl failed to recognize the rat β_2_AR in HEK 293 cells following transient transfection with rat β_2_AR cDNA (Figure 1C and 1D). To confirm that the rat β_2_AR was expressed in HEK 293 cells following transient transfection, ligand binding assays were performed using the β_2_AR antagonist [^3^H]DHA. Transiently transfected cells expressed (2.5 ± 0.5) × 10^6 ^receptors/cell, whereas untransfected HEK293 cells expressed 897 ± 558 receptors/cell. Taken together, these results indicate that Ab-Bethyl specifically recognizes human but not rat β_2_AR.

### Ab-sc570 Specifically Recognizes Rat β_2_AR in HEK 293 Cells

**Figure 2:**
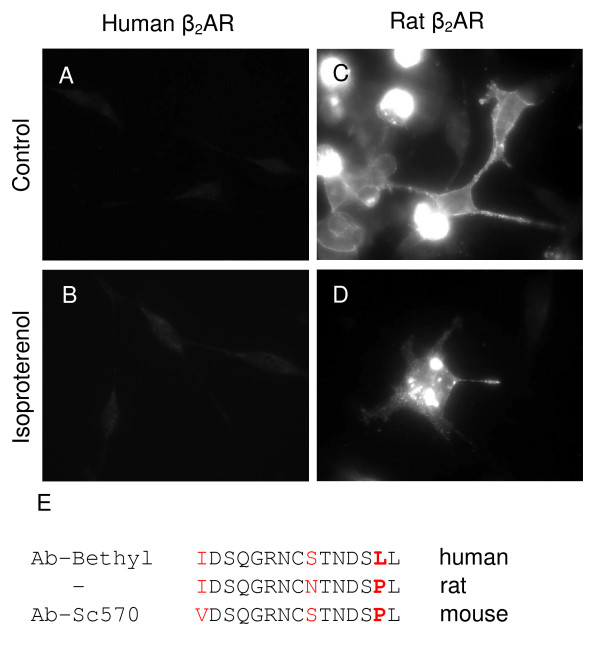
**Ab-sc570 Specifically Recognizes Rat β_2_AR in HEK 293 Cells**. HEK 293 cells stably expressing human β_2_AR (A, B) and HEK 293 cells transiently expressing rat β_2_AR (C, D) were either untreated (A, C) or treated (B, D) with isoproterenol for 4.5 h in parallel, fixed and processed for microscopy (Axioskop 2 plus epifluorescent microscope). (E) Sequence comparison of the last 15 amino acids of the human, rat and mouse β_2_AR.

To study β_2_AR trafficking in rat cells, either *in vitro *or *in vivo*, an antibody is needed that is capable of recognizing rat β_2_AR. Such an antibody might prove useful for localizing native β_2_AR in rat lung tissue and in primary cultures of rat airway epithelial and smooth muscle cells. Ab-sc570 antibody was developed against the C-terminal domain of the mouse β_2_AR which is 86.7% identical to rat β_2_AR. Therefore, Ab-sc570 was tested for recognition of rat β_2_AR by indirect immunofluorescence analysis in human cells. HEK 293 cells were transiently transfected with a plasmid encoding the rat β_2_AR cDNA (Figure 2C,D). In untreated transfected cells, bright cell surface staining was observed (Figure 2C). In cells treated with isoproterenol, the staining was concentrated in intracellular structures indicative of internalization of the receptors in response to agonist (Figure 2D). Ab-sc570 antibody did not recognize human β_2_AR in HEK 293 cells (Figure 2A,B). A comparison of the last 15 amino acids of rat, mouse and human β_2_AR (Figure 2E) reveals that the penultimate amino acid must account for the difference in recognition. In the human β_2_AR, the penultimate amino acid is hydrophobic leucine, whereas in the rat and mouse receptor it is proline. Since proline is an imino acid, the backbone geometry at the penultimate position might vary between rat/mouse and human homologs, which could be a local conformational variation. This difference appears to account for the recognition specificity of the rat and human β_2_AR by Ab-sc570 and Ab-Bethyl, respectively.

### Ab-13989 Specifically Recognizes Human and Rat β_2_AR in HEK 293 Cells

**Figure 3:**
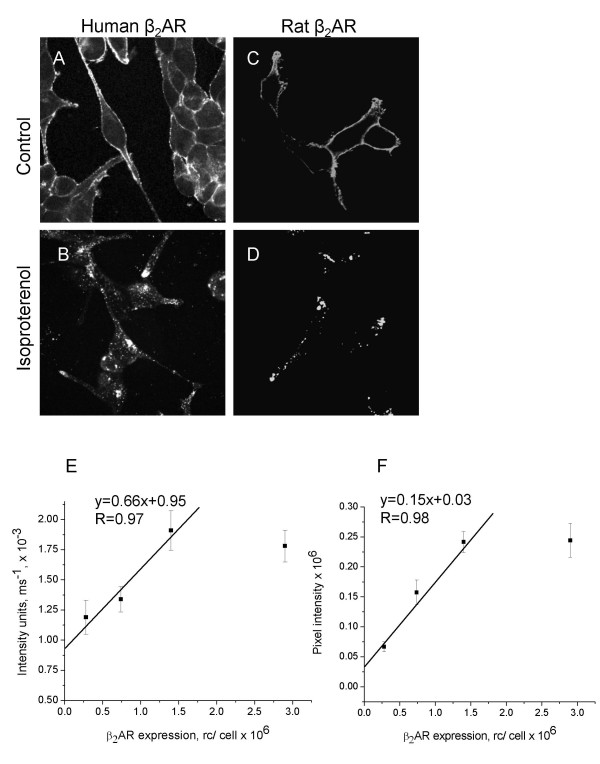
**Ab-13989 Specifically Recognizes Human and Rat β_2_AR in HEK 293 Cells**. HEK 293 cells stably expressing human β_2_AR (A, B) and HEK 293 cells transiently expressing rat β_2_AR (C, D) were either untreated (A, C) or treated (B, D) with isoproterenol for 4.5 h in parallel, fixed and processed for microscopy using a LSM510 confocal microscope. HEK 293 cell lines expressing different levels of human β_2_AR were processed and analyzed by wide field (E) and confocal (F) microscopy to establish the range over which receptor number and fluorescence signal intensity was linear.

Ab-13989 was raised against the large C-terminal domain (78 amino acids) of the human β_2_AR (Table 1). Given that the immunogen is large and that there is a high degree of amino acid conservation over the region between human and rat β_2_AR (73% identity, 79% similarity), we anticipated that this antibody would recognize both the rat and human receptors. Indeed, when tested in transfected HEK 293 cells, Ab-13989 recognized both rat and human β_2_AR (Figure 3).

We conducted semi-quantitative studies to define a linear range for detecting human β_2_AR using Ab-13989 on four HEK 293 cell lines stably expressing different levels of the β_2_AR ranging from 280,000 to 2,900,000 receptors/cell. Samples were analyzed by both wide field and confocal epifluorescence microscopy. For wide field microscopy, optimal exposure times for image acquisition were determined by software. Low signal intensities required longer exposure times whereas high signal intensities required shorter exposure time. Therefore, an arbitrary intensity unit was defined as the inverse of the exposure time. These results are plotted in Figure 3E and show a linear relationship between receptor number and staining intensity (R = 0.97) from ~280,000 to ~1,400,000 receptors per cell. Above ~1,400,000 receptors per cell, the signal plateaued (probably from quenching due to the interfilter effect), so this value was not used to calculate the correlation coefficient. For confocal microscope analysis, images were taken under identical detection conditions and the integrated signal intensity measured on a cell by cell basis. Results were essentially identical to those using the wide field microscope with a correlation coefficient of 0.98 (Figure 3F).

### Localization of the β_2_AR in Primary Cultures of Rat Airway Smooth Muscle and Rat Airway Epithelial Cells

**Figure 4:**
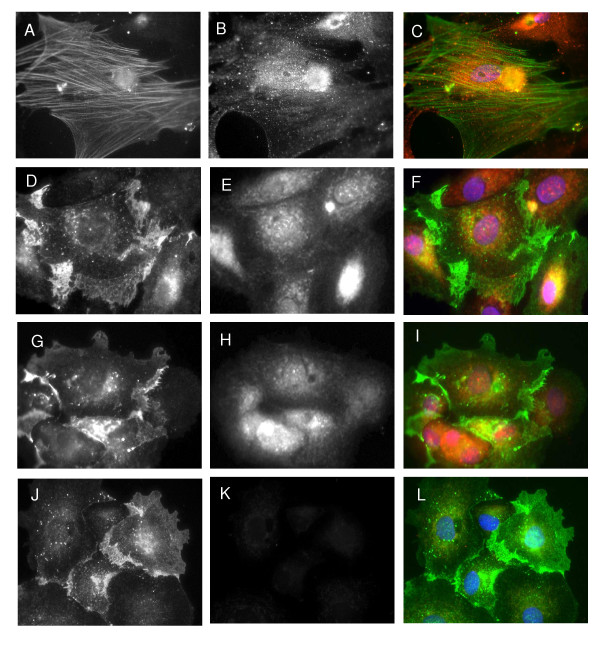
**Localization of the β_2_AR in Primary Cultures of Rat Airway Smooth Muscle and Rat Airway Epithelial Cells**. Primary cultures of rat ASM cells were derived from tracheal explants. Cells were fixed and double-labeled with β_2_AR (Ab-13989) (B) and anti-smooth muscle α-actin antibodies (A). C – merged image. Airway epithelial cells were harvested from rat lungs, fixed and double-labeled with β_2_AR (Ab-13989, E and Ab-sc570, H) and E-cadherin antibodies (D, G, and J). Ab-13989 and Ab-sc570 demonstrated a similar pattern of staining in primary cultures (E and H). Preincubation of Ab-sc570 with neutralizing peptide (sc570p) abrogated the staining (K). Panels F, I, L are merged images.

The majority of the studies on the β_2_AR have been performed using recombinant epitope- and fluorescent-tagged proteins [18-22]. However, relatively little is known about localization and regulation of endogenous β_2_AR. One study reported expression of β_2_AR in alveolar epithelium in paraffin embedded lung tissue [23]. Given the importance of β-agonists in the management of asthma, we sought to use Ab-sc570 and Ab-139898 in indirect immunofluorescence assays with primary cultures of rat airway epithelial and smooth muscle cells to localize native rat β_2_AR. We reasoned that the use of 2 distinct antibodies recognizing rat β_2_AR would provide a robust control for potential nonspecific binding of the antibodies. In addition, we used a competing peptide for Ab-sc570 as an additional specificity control. Cell-type specificity of the cultures was confirmed using anti-α-smooth muscle actin (an actin isoform typical of smooth muscle cells [24]) as a marker for smooth muscle cells and E-cadherin (a transmembrane glycoprotein localized in adherent junctions of epithelial cells [25,26]) as a marker for epithelial cells. Alpha-smooth muscle actin staining was localized on microfilament fibers in more than 80% of the cells in a preparation generated by outgrowth from denuded rat trachea (Figure 4A). E-cadherin staining was abundant in areas where epithelial cells were in close apposition (Figure 4D,G and 4J). Both Ab-sc570 and Ab-13989 stained primary cultures of rat airway smooth muscle and epithelial cells. However, compared with studies using HEK 293 cells that over-express the β_2_AR, native rat β_2_AR demonstrated a prominent intracellular distribution with a relative reduction in staining localized on the cell surface (Figures 4B,E, and 4H). We carefully analyzed images derived from rat primary cultures to define the fraction of staining that was intracellular. The analysis indicated that 43.7 ± 9.9% of the total signal for β_2_AR was intracellular in primary cultures of rat airway epithelial cells. By contrast, intracellular staining accounted for only 9.4 ± 5.8% staining in transfected HEK 293 cells. We also defined predominant plasma membrane localization (86.1 ± 6.3%) for E-cadherin in rat airway epithelial cells. These results show that a significant fraction of the native rat receptor was localized intracellularly. Furthermore, the patterns of staining for the β_2_AR in rat primary cultures using two distinct antibodies raised against different portions of the β_2_AR (Table 1) were remarkably similar (Figure 4B,E, and 4H) indicating that the signal is likely specific. In addition, preincubation of Ab-sc570 with a 5-fold mass excess of neutralizing peptide completely abrogated the staining (Figure 4K). Thus, it appears that a significant proportion of rat airway β_2_AR are inside the cell rather than on the surface. This might be explained by differences in the level of expression of the receptors between the two systems. The HEK 293 cells we used for antibody characterization expressed 32,764 ± 2,173 fmol receptors/mg cellular protein (which corresponded to 1.36 × 10^6 ^receptors/cell) – approximately 95 times higher than the level in primary cultures of rat airway epithelial cells (345 ± 8 fmol receptor/mg protein). The prominent cell surface expression noted in HEK 293 cells could be a consequence of saturating the mechanisms responsible for constitutive internalization or for intracellular retention of β_2_AR.

## Conclusion

The β_2_AR is an important target for medications used to treat respiratory and cardiovascular diseases. The development of tolerance to repetitive doses of β-agonist is a significant clinical problem. Therefore, studies on the molecular mechanism regulating β_2_AR activity after treatment and in different physiologic conditions are of importance in designing better therapies for treatment. Immunofluorescence and immunohistochemical methods are of value in studying trafficking and regulation of the β_2_AR because they can be used in the context of the whole tissue. In this study, we evaluated six β_2_AR antibodies developed against different portions of the β_2_AR. We identified one antibody that specifically recognized human β_2_AR, one antibody that specifically recognized rat β_2_AR, and one antibody capable of recognizing both rat and human β_2_AR. In HEK 293 cells, both rat and human β_2_AR were localized to the cell surface in untreated cells following transfection and moved into an intracellular compartment within a few hours of treatment with the β-agonist isoproterenol. Although these findings are in complete agreement with previous studies performed using tagged β_2_AR, results of an analysis of the localization of endogenous rat airway β_2_AR were not. We made the novel observation that almost half of the endogenous rat β_2_ARs are located in an intracellular compartment instead of being largely restricted to the plasma membrane. Specificity controls, and especially the fact that the pattern of staining was identical using two different antibodies raised against different potions of the receptor, support our conclusion.

It is possible that receptor localization in HEK 293 cells may be altered as a consequence of expressing receptors at a level 100 times higher than normal. Saturation of the mechanisms for constitutive internalization and intracellular retention of β_2_AR may account for the prominent cell surface expression consistently noted in HEK 293 cells. Alternatively, there could be cell-specific differences in internalization mechanisms that are independent of receptor number. In either case, the significant differences in receptor localization compromise the utility of using tagged receptors in HEK 293 cells to define receptor trafficking pathways relevant to the problem of β-agonist tolerance in airway smooth muscle or epithelial cells.

Our results demonstrating that almost half of the β_2_AR in cultures of primary airway cells are located inside the cells underscores the need for future studies assessing the location and trafficking of endogenous β_2_AR in airway smooth muscle and epithelium. The antibodies that we have characterized now provide the tools needed for such studies.

## Competing interests

The authors declare that they have no competing interests.

## Authors' contributions

YAK performed the studies and wrote the first draft of the manuscript. TWF generated the primary rat airway and smooth muscle cells used for the studies. BJS provided us with cell line stably expressing human β_2_AR. SMJ, LEC and RCK conceived the studies, secured funding support, participated in the design and troubleshooting of the experiments and in the revision of the manuscript.
